# Genome-Wide Chromatin Immunoprecipitation Sequencing Analysis Shows that WhiB Is a Transcription Factor That Cocontrols Its Regulon with WhiA To Initiate Developmental Cell Division in *Streptomyces*

**DOI:** 10.1128/mBio.00523-16

**Published:** 2016-04-19

**Authors:** Matthew J. Bush, Govind Chandra, Maureen J. Bibb, Kim C. Findlay, Mark J. Buttner

**Affiliations:** a Department of Molecular Microbiology, John Innes Centre, Norwich, United Kingdom; b Department of Cell and Developmental Biology, John Innes Centre, Norwich, United Kingdom

## Abstract

WhiB is the founding member of a family of proteins (the WhiB-like [Wbl] family) that carry a [4Fe-4S] iron-sulfur cluster and play key roles in diverse aspects of the biology of actinomycetes, including pathogenesis, antibiotic resistance, and the control of development. In *Streptomyces*, WhiB is essential for the process of developmentally controlled cell division that leads to sporulation. The biochemical function of Wbl proteins has been controversial; here, we set out to determine unambiguously if WhiB functions as a transcription factor using chromatin immunoprecipitation sequencing (ChIP-seq) in *Streptomyces venezuelae*. In the first demonstration of *in vivo* genome-wide Wbl binding, we showed that WhiB regulates the expression of key genes required for sporulation by binding upstream of ~240 transcription units. Strikingly, the WhiB regulon is identical to the previously characterized WhiA regulon, providing an explanation for the identical phenotypes of *whiA* and *whiB* mutants. Using ChIP-seq, we demonstrated that *in vivo* DNA binding by WhiA depends on WhiB and vice versa, showing that WhiA and WhiB function cooperatively to control expression of a common set of WhiAB target genes. Finally, we show that mutation of the cysteine residues that coordinate the [4Fe-4S] cluster in WhiB prevents DNA binding by both WhiB and WhiA *in vivo*.

## IMPORTANCE

Despite the central importance of WhiB-like (Wbl) proteins in actinomycete biology, a conclusive demonstration of their biochemical function has been elusive, and they have been difficult to study, particularly *in vitro*, largely because they carry an oxygen-sensitive [4Fe-4S] cluster. Here we used genome-wide ChIP-seq to investigate the function of *Streptomyces* WhiB, the founding member of the Wbl family. The advantage of this approach is that the oxygen sensitivity of the [4Fe-4S] cluster becomes irrelevant once the protein has been cross-linked to DNA *in vivo*. Our data provide the most compelling *in vivo* evidence to date that WhiB, and, by extension, probably all Wbl proteins, function as transcription factors. Further, we show that WhiB does not act independently but rather coregulates its regulon of sporulation genes with a partner transcription factor, WhiA.

## INTRODUCTION

The complex life cycle of streptomycetes involves two distinct filamentous cell forms, the growing or vegetative hyphae and the reproductive or aerial hyphae, which differentiate into long chains of spores. This life cycle involves two major developmental transitions, controlled by two separate classes of developmental regulators (1–3). In the first transition, the reproductive hyphae grow away from the substrate mycelium and into the air to form the aerial mycelium, which gives the colonies a characteristic fuzzy appearance. The erection of the aerial mycelium is controlled by the Bld (Bald) regulators, so called because mutations in the genes encoding these regulators give rise to colonies with a shiny “bald” appearance. c-di-GMP plays a critical role in controlling progression through the developmental cycle, with the master repressor BldD directly mediating the effects of c-di-GMP signaling on the entire regulatory cascade (3, 4). In the second transition, the reproductive hyphae cease tip growth and undergo a massive cell division event involving the synchronous formation of dozens of sporulation septa that divide the multigenomic tip cell into a long chain of unigenomic prespore compartments (5, 6). These cylindrical compartments then differentiate into rounded, mature spores. The differentiation of aerial hyphae into spores involves the profound reorganization of the growth, morphology, and physiology of the cells. This process is controlled by the Whi (White) regulators, so called because mutations in the genes encoding these regulators prevent the synthesis of the characteristic polyketide pigment associated with mature spores, giving rise to white colonies.

The work described here focuses on the sporulation regulatory proteins WhiA and WhiB, the only Whi regulators that are absolutely required for sporulation septation in *Streptomyces*. *Streptomyces coelicolor* *whiA* and *whiB* mutants fail to halt aerial growth, to initiate the synchronous septation event, or to partition their chromosomes. Instead, the cells keep growing, producing long aerial hyphae that lack sporulation septa and contain uncondensed DNA (7, 8). These identical phenotypes have led to the suggestion that WhiA and WhiB might function together to control a distinct pathway within the sporulation regulatory network (3, 7, 8).

WhiA and WhiB are of interest not only because they play key roles in *Streptomyces* developmental biology but also because they are the founding members of two highly unusual families of proteins, both of which have proved difficult to study. The crystal structure of WhiA reveals a large N-terminal domain related to a class of eukaryotic homing endonucleases. This domain lacks the residues required for catalysis and displays an altered surface charge, suggesting that it does not cleave or bind DNA (9, 10). Instead, DNA recognition is mediated by a small C-terminal helix-turn-helix (HTH) DNA-binding domain in WhiA that is absent from classical homing endonucleases, and the function of the homing endonuclease-like domain remains unknown (10–12), Interestingly, members of the WhiA family are found throughout the Gram-positive bacteria, including nonactinomycetes and bacteria that do not sporulate. In *Bacillus subtilis*, WhiA/YvcL binding to DNA facilitates its localization to the nucleoid, where it is proposed to have a direct biological role in regulating cell division (13). This suggests that not all WhiA family members function as transcription factors.

WhiB is the founding member of a class of actinomycete-specific proteins known as the Wbl (WhiB-like) family (14–16). Wbl proteins have been shown to contain four conserved cysteines that form an oxygen- and nitric oxide-sensitive [4Fe-4S] cluster (17–21). The *S. coelicolor* chromosome encodes 11 Wbl proteins, three of which have been shown to have important functions in development. Besides WhiB, WblA plays a role in the formation of aerial hyphae (22–24) and WhiD is required for prespore maturation (25). Outside the *Streptomyces* genus, Wbl proteins in *Corynebacterium diphtheriae* and *Mycobacterium tuberculosis* have been implicated in key stages of pathogenesis and in antibiotic resistance (26). The exact biochemical function of Wbl proteins has been controversial and difficult to analyze experimentally, in large part because of the presence of the oxygen-sensitive [4Fe-4S] cluster. While some studies have claimed alternative roles for Wbl proteins (27), they have long been suggested to function as transcription factors (15, 16). Consistent with this prediction, several studies have presented results from electrophoretic mobility shift assays (EMSAs) using mycobacterial and streptomycete Wbl proteins that are consistent with weak binding to specific promoters (20, 26, 28–30). However, more-robust *in vitro* evidence of site-specific DNA binding such as footprinting is still absent, and, crucially, no Wbl protein has been shown to bind DNA in a site-specific manner *in vivo* by genome-wide chromatin immunoprecipitation sequencing (ChIP-seq) analysis.

Until recently, the study of *Streptomyces* development has been hampered because the classical model species, *S. coelicolor*, sporulates only on solid medium and ~95% of the colony is found within the agar as vegetative mycelium, with the aerial hyphae representing only the remaining ~5% of the total biomass. This makes the detection of subtle changes in the transcriptome associated with sporulation difficult and the application of other global techniques such as ChIP-seq to development often impractical. In contrast, *Streptomyces venezuelae* sporulates synchronously and to near-completion in liquid culture (3, 31, 32), greatly facilitating the application of global “omics” and cell biological techniques to the study of differentiation. Using ChIP-seq analysis, this new model organism has now been successfully exploited to identify the genes under the control of the developmental regulators σ^BldN^ (33), BldM, WhiI (34), and WhiA (12).

We previously showed by ChIP-seq analysis that the developmental regulator WhiA directly activates the expression of genes required for sporulation septation and chromosome segregation at the onset of sporulation in *S. venezuelae* (12). Here, using a similar approach, we set out to determine how WhiB mediates its effects on developmentally controlled cell division. We show that WhiB and WhiA directly coregulate the same set of genes, that WhiA is required for *in vivo* DNA binding by WhiB and vice versa, and that mutation of the cysteine residues that coordinate the [4Fe-4S] cluster in WhiB prevents DNA binding by both WhiB and WhiA. These findings provide an explanation for the identical phenotypes of *whiA* and *whiB* mutants.

## RESULTS AND DISCUSSION

### WhiA and WhiB control the same stage of *Streptomyces* development.

**FIG 1:**
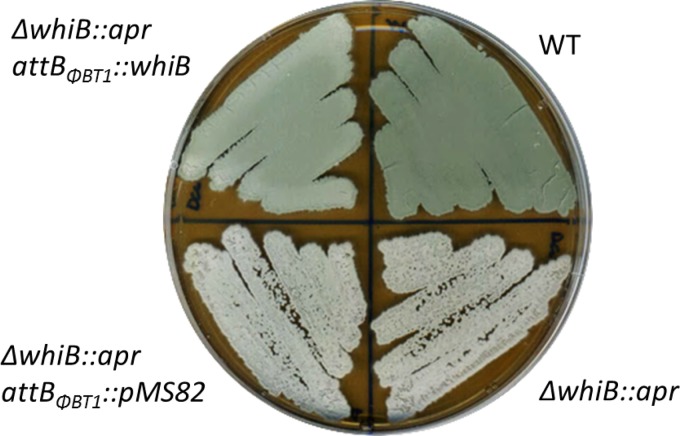
Deletion of *whiB* prevents sporulation. Shown are the phenotypes of wild-type *S. venezuelae* (WT), the constructed Δ*whiB*::*apr* SV7 null mutant (Δ*whiB*::*apr*), SV7 carrying the empty pMS82 vector (Δ*whiB*::*apr attB_ΦBT1_*::pMS82), and the complemented strain SV7/pIJ6761 (Δ*whiB*::*apr attB*_Φ*BT1*_::*whiB*). Strains were grown on MYM solid medium and photographed after 4 days.

**FIG 2:**
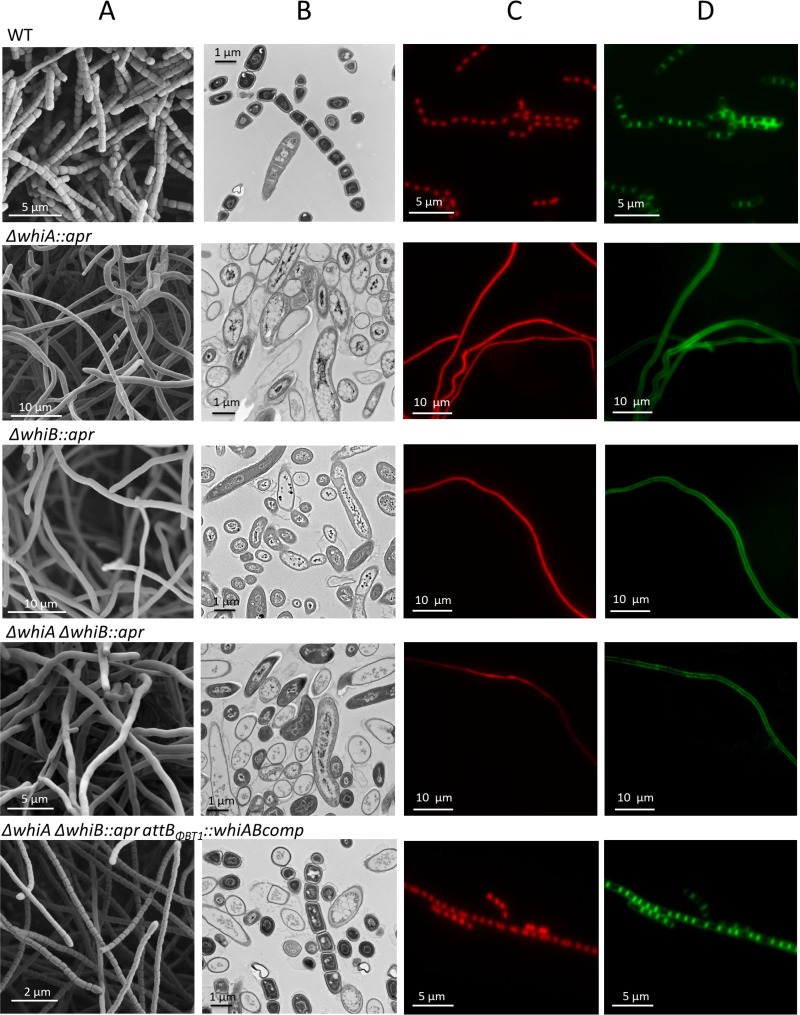
*whiA* and *whiB* mutants have identical phenotypes. The data compare the phenotypes of wild-type *S. venezuelae* (WT), the Δ*whiA* SV11 null mutant (Δ*whiA*::*apr*) (12), the constructed Δ*whiB* SV7 null mutant (Δ*whiB*::*apr*), the Δ*whiA* Δ*whiB* SV51 double mutant (Δ*whiA* Δ*whiB*::*apr*), and the SV51/pIJ10604 complemented strain (Δ*whiA* Δ*whiB*::*apr attB*_Φ*BT1*_::*whiABcomp*). Strains were examined by scanning electron microscopy (A), transmission electron microscopy (B), and fluorescence microscopy after staining DNA and the cell wall with 25 µg/ml propidium iodide (C) and 50 µg/ml wheat germ agglutinin (WGA) Alexa Fluor 488 (D), respectively. All of the hyphae shown are aerial hyphae. Strains were grown on MYM solid medium for 2 days before fluorescence microscopy and 4 days before electron microscopy. Scale bars are as indicated.

An *S. venezuelae* ΔwhiB::*apr* mutant was constructed and its phenotype analyzed. The *whiB* mutant failed to synthesize the green pigment characteristic of mature *S. venezuelae* spores, instead appearing white (Fig. 1). Scanning electron microscopy (SEM) and DNA staining by propidium iodide (PI) showed that the *S. venezuelae* whiB mutant forms long, extended aerial hyphae that fail to lay down sporulation septa or to segregate their chromosomes (Fig. 2). This is directly comparable to the phenotype of *S. coelicolor* *whiB* mutants (7), although it should be noted that *S. venezuelae* forms straight aerial hyphae rather than the coiled aerial hyphae found in *S. coelicolor* and that this difference is reflected in the phenotype of the *whiB* mutants, as seen in other *whi* mutants, including *whiA* (12). Normal sporulation was restored to the *S. venezuelae* *whiB* mutant by introducing a single copy of the wild-type *whiB* gene under the control of its native promoter, expressed in *trans* from the ΦBT1 integration site (Fig. 1). Thus, WhiB appears to play similar roles in *S. venezuelae* and *S. coelicolor.*

The phenotype of the *S. venezuelae whiB* mutant was indistinguishable from the phenotype of the *S. venezuelae whiA* mutant (12), as was previously observed in *S. coelicolor* (7, 8). To examine this further and to facilitate subsequent studies, we constructed an *S. venezuelae whiAB* double mutant. To begin with, a *whiA* mutant strain was made using the I-SceI Meganuclease system (35). The resulting strain was unmarked, showed a phenotype identical to that of the previously constructed *ΔwhiA*::*apr* strain (12), and could be complemented by expressing a wild-type copy of *whiA* from the ΦBT1 integration site (data not shown). Next, the *ΔwhiB*::*apr* allele was introduced into the markerless *whiA* mutant by phage SV1-mediated generalized transduction (36). The resulting *whiAB* double mutant displayed the same phenotype as the *whiA* and *whiB* single mutants (Fig. 2) and could be complemented by introducing single copies of both *whiA* and *whiB* under the control of their native promoters but not by either gene individually (see Fig. S1 in the supplemental material). In addition, the *whiA* and *whiB* genes were individually overexpressed using the strong, constitutive ermE* promoter. Overexpressing WhiA complemented the *whiA* mutant but had no effect on the phenotypes of the *whiB* or *whiAB* mutants (see Fig. S2). Similarly, overexpressing WhiB restored sporulation to a *whiB* mutant but had no effect on the phenotypes of the *whiA* or *whiAB* mutants (see Fig. S2). Thus, neither WhiA nor WhiB is able to promote sporulation independently of the other.

### Defining the WhiB regulon.

The benefit of using ChIP-seq to determine if WhiB functions as a transcription factor is that any concerns relating to the oxygen sensitivity of the [4Fe-4S] cluster become irrelevant once the protein has been cross-linked to DNA *in vivo*. Therefore, we conducted two separate ChIP-seq experiments. In the first experiment, an anti-WhiB polyclonal antibody was raised and used for ChIP-seq analysis with cultures of wild-type *S. venezuelae*, with the congenic *ΔwhiB*::*apr* mutant serving as the negative control. In the second experiment, an anti-FLAG antibody was employed to immunoprecipitate a FLAG-tagged version of WhiB. The latter (preferred) approach required the construction of a strain of *S. venezuelae* that lacked *whiB* at its native locus but expressed an N-terminally triple-FLAG (3×FLAG)-tagged version of WhiB, under the control of its native promoter, from the ΦBT1 integration site. The FLAG-tagged allele was engineered such that WhiB was separated from the FLAG tag by a flexible [Gly_4_Ser]_3_ linker (as previously used in WhiA ChIP-seq analysis [12]). Importantly, the *ΔwhiB*::*apr* mutant carrying the FLAG-tagged *whiB* allele sporulated normally both on MYM agar and in MYM liquid medium, showing that the FLAG-tagged version of WhiB was fully functional (Fig. S3A in the supplemental material and data not shown). Wild-type *S. venezuelae* served as the negative control in the anti-FLAG-based ChIP-seq experiment.

In both the anti-WhiB and anti-FLAG experiments, ChIP-seq was conducted at the onset of sporulation in liquid culture. In addition to the negative controls (the *whiB* mutant for the anti-WhiB experiment and the wild-type strain for the anti-FLAG experiment), total (nonimmunoprecipitated) input DNA was also subjected to deep sequencing. This additional control allows normalization of nonuniform shearing of the chromosome (12, 37). Using a *P* value of <10^−4^ as the threshold for significance, a total of 236 peaks were detected in one or both of the test strains at the time point selected (see Table S1A and Fig. S3B in the supplemental material). Of these, 44 were located more than 300 bp upstream of the nearest annotated start codon (see Table S1A) and were not analyzed further. Using the same threshold for significance, 19 peaks were identified in the wild-type anti-FLAG negative-control experiment (see Fig. S4 and Table S1B) and 1 peak (within the *sven0993* coding region) was identified in the *whiB* mutant anti-WhiB negative-control experiment (see Table S1B), and these peaks were excluded from subsequent analysis. Importantly, the vast majority of peaks that fell above the threshold were identified in both the anti-FLAG and anti-WhiB experiments. Indeed, visual inspection of the raw data revealed an excellent correlation between the two data sets, with the genome-wide enrichment profiles closely resembling one another and the individual ChIP-seq peaks overlaying almost perfectly (see Fig. S4). These data provide the most compelling *in vivo* evidence to date that WhiB, and, by extension, probably all Wbl proteins, functions as transcription factors.

### WhiA and WhiB coregulate the same set of genes.

Strikingly, analysis of the WhiB target genes revealed a nearly complete overlap of the WhiA and WhiB regulons. Of the 192 WhiB ChIP-seq peaks located less than 300 bp upstream of the nearest annotated start codon, 103 were also identified in the previous study of the WhiA regulon (12), using a *P* value of <10^−4^ as the threshold for significance. Closer inspection revealed that the vast majority of the remaining WhiA or WhiB targets were in fact present in both data sets but that either the WhiA or WhiB peak fell just outside the *P* threshold of <10^−4^ (data not shown). Furthermore, there was a clear correlation between the significance values for individual targets in the WhiA and WhiB datasets. In other words, the most strongly enriched targets in one data set were also highly significant in the other and, conversely, those which showed only modest enrichment in one data set also showed lower significance in the other. This strongly suggests that WhiA and WhiB control the same set of genes. Any apparent differences seen between the WhiA and WhiB regulons likely reflect low-level heterogeneity between individual cultures in experiments that were performed independently of one another, years apart. It therefore appears that there are few, if any, uniquely WhiA-specific or WhiB-specific targets.

**FIG 3:**
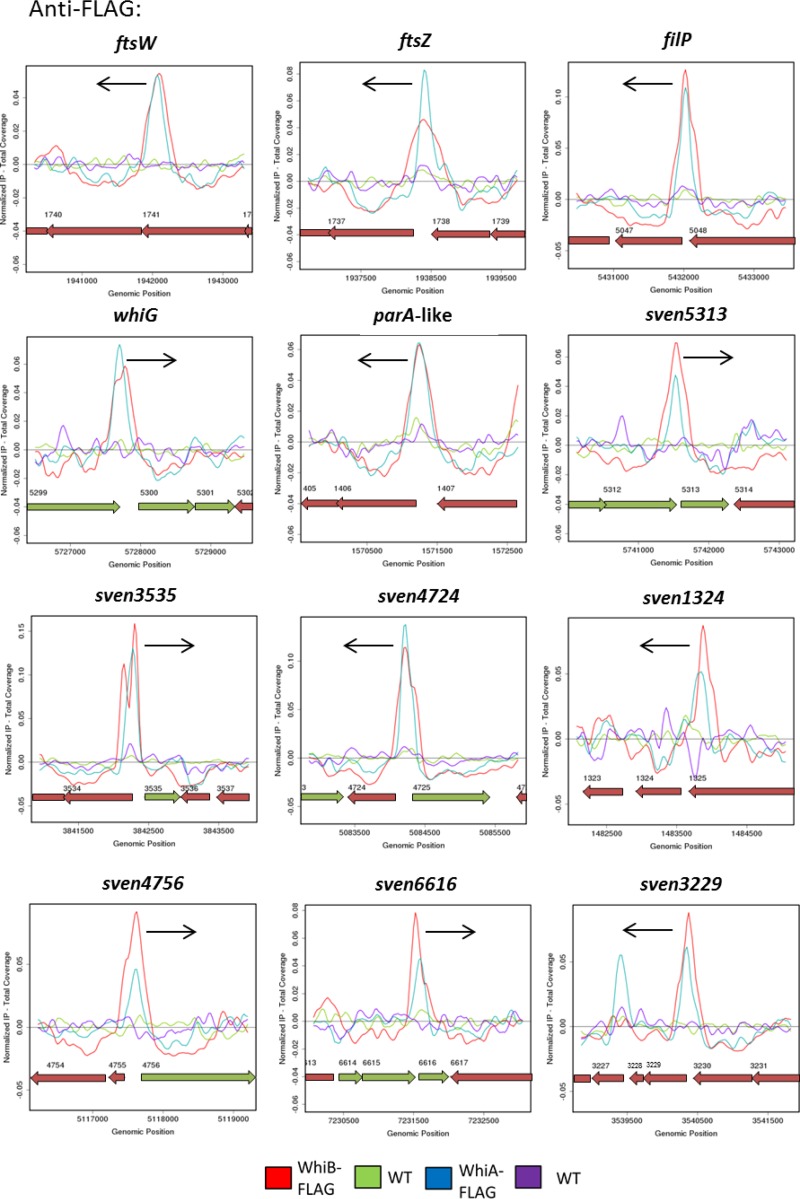
WhiA and WhiB have a shared regulon. The data compare anti-FLAG ChIP-seq results for WhiA and WhiB. ChIP traces are shown for 12 selected WhiA and WhiB target genes: *ftsW*, *ftsZ*, *filP*, *whiG*, *sven1406*, *sven5313*, *sven3535*, *sven4724*, *sven1324*, *sven4756*, *sven6616*, and *sven3229*. Color coding of the ChIP samples is as follows: 3×FLAG-[Gly_4_Ser]_3_-WhiB strain (WhiB-FLAG), red; corresponding *S. venezuelae* wild-type anti-FLAG negative control (WT), green; 3×FLAG-[Gly_4_Ser]_3_-WhiA strain (WhiA-FLAG), blue; and corresponding *S. venezuelae* wild-type anti-FLAG negative control (WT), purple. Plots span approximately 3 kb of DNA sequence. Genes running left to right are shown in green, and genes running right to left are shown in red. The black arrow indicates the gene subject to WhiA and WhiB regulation. The arrangement of the 12 panels mirrors that in Fig. 4.

Further comparison of the WhiA and WhiB anti-FLAG ChIP-seq datasets showed that the WhiA and WhiB peaks centered on the same genomic position for the vast majority of targets (Fig. 3). Although standard ChIP-seq analysis does not provide high-level resolution of DNA-binding sites, these results suggest that WhiA and WhiB bind to DNA in close proximity. Consistent with these results, when we subjected polyclonal or anti-FLAG WhiB ChIP samples to anti-WhiA Western blot analyses, WhiA was readily detected (see Fig. S5 in the supplemental material).

**FIG 4:**
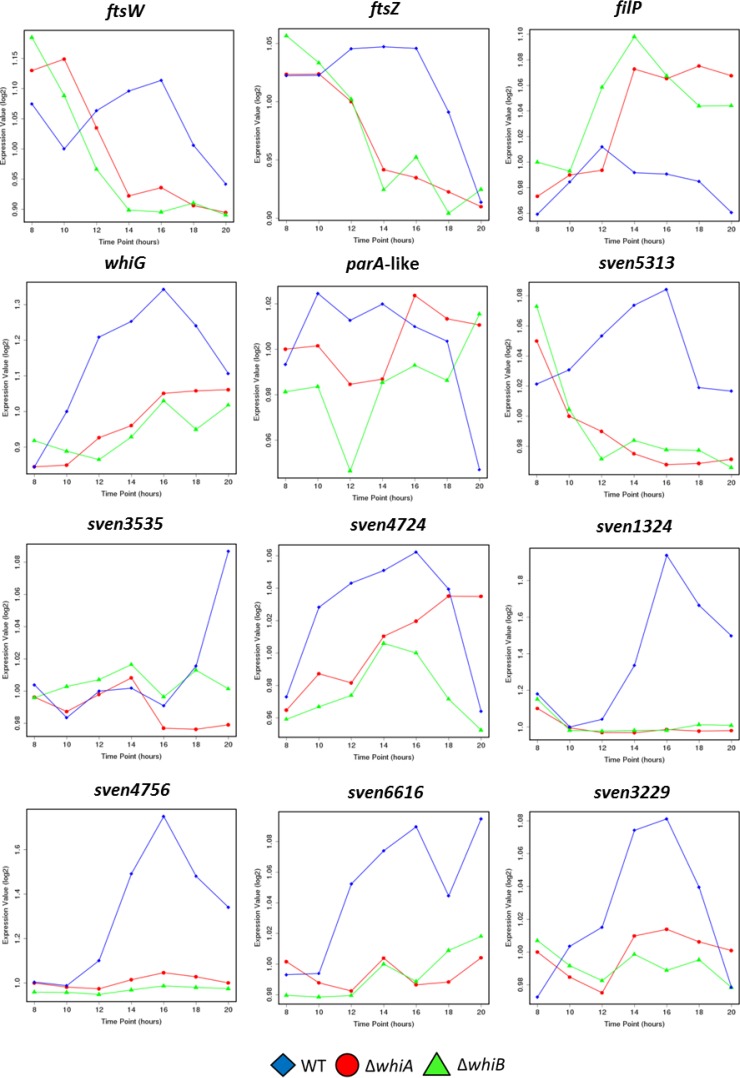
WhiA and WhiB targets depend on both *whiA* and *whiB* for their expression. Data represent results of microarray transcriptional profiling for 12 selected WhiA and WhiB target genes (*ftsW*, *ftsZ*, *filP*, *whiG*, *sven1406*, *sven5313*, *sven3535*, *sven4724*, *sven1324*, *sven4756*, *sven6616*, and *sven3229*) during submerged sporulation in wild-type *S. venezuelae* (blue diamonds); the congenic *whiA* mutant, SV11 (red circles); and the congenic *whiB* mutant, SV7 (green triangles). In each panel, the *x* axis indicates the age of the culture in hours, and the *y* axis indicates the per-gene normalized transcript abundance (log_2_). For the wild type, 10 to 14 h corresponds to vegetative growth, 14 to 16 h corresponds to the onset of sporulation (fragmentation), and 16 h and beyond corresponds to sporulation. The arrangement of the 12 panels mirrors that in Fig. 3.

We previously showed how WhiA influences the expression of its target genes by subjecting wild-type *S. venezuelae* and its congenic *ΔwhiA* mutant to transcriptional profiling throughout development in a submerged culture (12). In parallel with those experiments, we determined how WhiB influences genome-wide expression by subjecting the congenic *ΔwhiB* mutant to time-resolved, genome-wide transcriptional profiling during vegetative growth and sporulation, again in a submerged culture. Strains were grown under the same conditions as were used for the WhiB ChIP-seq experiments. As in our previous *whiA* study (12), RNA samples were prepared at 2-h intervals from 8 to 20 h, by which time sporulation in the wild type was nearing completion, and after cDNA synthesis and labeling, samples were hybridized to Affymetrix DNA microarrays. Examination of the resulting transcriptional profiling data and comparison with the equivalent data generated from the congenic *ΔwhiA* mutant revealed notable similarities in the dependence of the WhiAB target genes on the WhiA and WhiB transcription factors. Indeed, many targets showed nearly identical transcriptional profiles in the *whiA* and *whiB* mutants (Fig. 4). These data also showed that, like WhiA, WhiB is bifunctional, acting as an activator at many target genes and as a repressor at many others (Fig. 4; see also Table S1A in the supplemental material).

### WhiAB and the arrest of aerial growth.

WhiB, like WhiA, functions to repress the transcription of *filP*, encoding a cytoskeletal protein that localizes close to the hyphal tips (Fig. 3 and 4) (38, 39). *Streptomyces* hyphae employ an *mreB*-independent method of apical growth directed by a complex of three coiled-coil proteins termed the polarisome, a structure that is found at all growing hyphal tips. DivIVA is the only polarisome component essential for viability, likely acting as a landmark protein to recruit the cell wall biosynthetic machinery and to select future branch sites (40, 41). The other two components, FilP and Scy, nonetheless also play key roles in polar growth by tip extension. Scy has been suggested to function as a molecular scaffold to assist the assembly of the other polarisome components, and recent evidence suggests that Scy mediates a link between the growth of the aerial hyphae and chromosome segregation into nascent prespores via recruitment of the partitioning protein ParA (42, 43). FilP localizes immediately behind DivIVA foci at the hyphal tips and has been shown to form net-like cytoskeletal structures with stress-bearing properties, suggesting that it provides mechanical support for the growing hyphal tip (38, 39). During *Streptomyces* differentiation, before sporulation can proceed, the aerial hyphae must arrest tip growth, and during this transition, the polarisome is disassembled (43–45). *whiA* and *whiB* mutants form abnormally long aerial hyphae, showing that the timely cessation of aerial growth that precedes sporulation septation in the wild type depends on the functions of both WhiA and WhiB (Fig. 2) (7, 8, 12). How this dependence is mediated is not known. However, the corepression of *filP* by WhiA and WhiB at the onset of sporulation appears to be an attractive potential component of the underlying mechanism.

### WhiAB proteins coactivate genes required for developmental cell division.

The ChIP-seq and transcriptional profiling data also reveal that, like WhiA, WhiB functions to activate the expression of genes encoding key components of the cell division machinery, including *ftsZ*, *ftsW*, and *ftsK* (Fig. 3 and 4; see also Table S1A in the supplemental material)*.* FtsZ, the bacterial homolog of mammalian β-tubulin, assembles into a contractile ring (the Z ring) on the inner surface of the cytoplasmic membrane at the future site of septum synthesis to initiate bacterial cell division. The Z ring constricts at the division site, directing the synthesis of the ingrowing cell wall annulus, and the Z ring is required to recruit other proteins involved in septum formation to the division site. Streptomycetes employ two distinct forms of cell division during their life cycle (3). During vegetative growth, *ftsZ* is required only to form infrequent vegetative cross-walls, which compartmentalize the substrate mycelium but do not lead to constriction or cell-cell separation. In contrast, 50 to 100 Z-rings are synchronously assembled in each reproductive hypha to direct the synthesis of the sporulation septa that create the prespore compartments (1). An increase in FtsZ protein levels is known to be a critical factor in the initiation sporulation, and this transition depends on the substantial upregulation of *ftsZ* expression from a developmentally regulated promoter (46). During vegetative growth, BldD−(c-di-GMP) represses expression of its regulon of sporulation genes, including *ftsZ* (4, 47). In addition, it is now clear that WhiA and WhiB function to activate *ftsZ* expression at the onset of sporulation, since expression of *ftsZ* is equally downregulated in *whiA* and *whiB* mutants (Fig. 3 and 4; see also Table S1A). Thus, coactivation by WhiAB, coupled with release from BldD−(c-di-GMP)-mediated repression, may provide the increase in FtsZ levels required to initiate sporulation septation. Similarly, WhiB and WhiA also function to activate transcription of *ftsW* and *ftsK* (Fig. 3 and 4; see also Table S1A). In *E. coli*, FtsW is required to recruit its cognate transpeptidase (FtsI/PBP3) to the division site and is also likely the lipid II “flippase” (48, 49). FtsK is as a DNA translocase, functioning to pump the terminal ends of the chromosomes away from the constricting septa during the final stages of sporulation (50). In summary, the results of the ChIP-seq and microarray transcriptional profiling analyses are consistent with a central role for WhiA and WhiB in cocontrolling the switch from aerial growth to the initiation of sporulation.

### WhiA and WhiB bind to target genes in a codependent manner.

**FIG 5:**
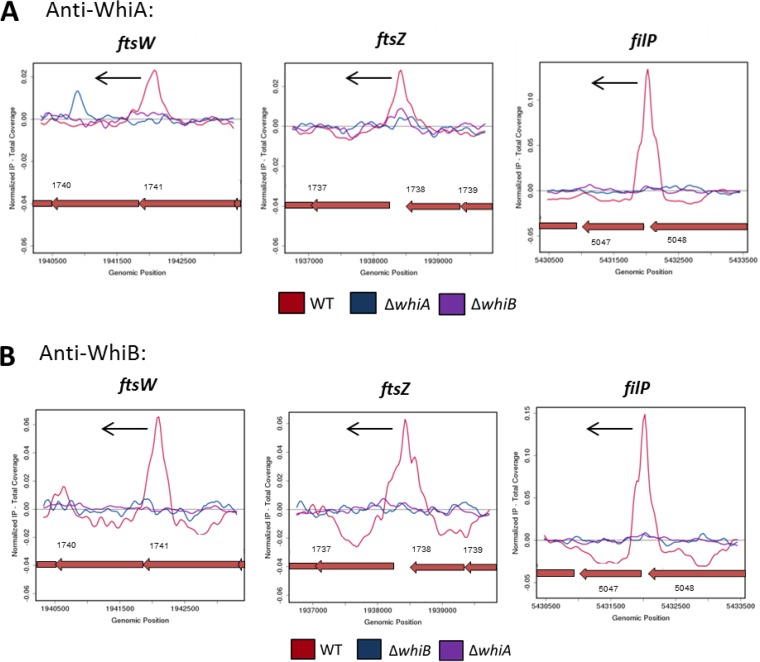
WhiB binding to its target promoters depends on WhiA and vice versa. ChIP-seq data for three representative WhiA and WhiB target genes, *ftsW*, *ftsZ*, and *filP*, are shown. (A) Anti-WhiA ChIP-seq in the presence and absence of WhiB. Color coding of the ChIP samples is as follows: *S. venezuelae* wild-type strain (WT), red; Δ*whiA* negative control (*ΔwhiA*), blue; *ΔwhiB* strain (*ΔwhiB*), purple. (B) Anti-WhiB ChIP-seq in the presence and absence of WhiA. Color coding of the ChIP samples is as follows: *S. venezuelae* wild-type strain (WT), red; *ΔwhiB* negative control (*ΔwhiB*), blue; *ΔwhiA* strain (*ΔwhiA*), purple. Plots span approximately 3 kb of DNA sequence. Genes running right to left are shown in red. The black arrow indicates the gene subject to WhiA and WhiB regulation.

To determine if WhiB binding to its target promoters depends on WhiA and vice versa, we conducted WhiA ChIP-seq analysis in a *whiB* mutant, and WhiB ChIP-seq analysis in a *whiA* mutant, using the wild type as a positive control. These experiments were conducted with the WhiB polyclonal antibody described above and the WhiA polyclonal antibody described in our previous study (12). The WhiA ChIP-seq peaks seen in the wild type were absent from the *whiB* mutant (Fig. 5A; see also Table S1C in the supplemental material), and, similarly, the WhiB ChIP-seq peaks seen in the wild type were absent from the *whiA* mutant (Fig. 5B; see also Table S1C). Importantly, Western blots revealed that the WhiA protein was readily detected in a *whiB* mutant and vice versa, showing that the lack of enrichment in these experiments was not due to protein instability (see Fig. S6)*.* Therefore, we conclude that, *in vivo*, WhiA and WhiB cannot bind to their target promoters independently of each other.

### Mutation of the cysteine residues that coordinate the WhiB [4Fe-4S] cluster prevents *in vivo* DNA binding by both WhiB and WhiA.

**FIG 6:**
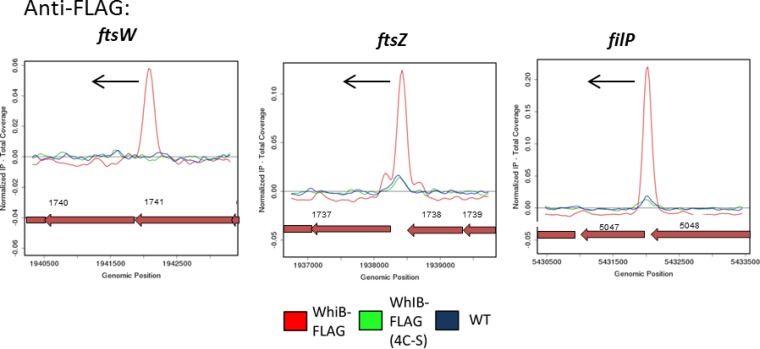
Mutation of the cysteine residues that coordinate the WhiB [4Fe-4S] cluster prevents DNA binding *in vivo*. Anti-FLAG ChIP-seq data for WhiB and WhiB (4C-S) are shown for three representative WhiA and WhiB target genes: *ftsW*, *ftsZ*, and *filP*. Color coding of the ChIP samples is as follows: 3×FLAG-[Gly_4_Ser]_3_-WhiB strain (WhiB-FLAG), red; 3×FLAG-[Gly_4_Ser]_3_-WhiB(4C-S) strain, green; corresponding *S. venezuelae* wild-type anti-FLAG negative control (WT), blue. Plots span approximately 3 kb of DNA sequence. Genes running right to left are shown in red. The black arrow indicates the gene subject to WhiA and WhiB regulation.

Wbl family members contain four conserved cysteine residues that bind a [4Fe-4S] cluster (17). These clusters may play an entirely structural role, but *in vitro* studies using WhiB1, WhiB3, and WhiD have shown that they are extremely sensitive to nitric oxide and somewhat sensitive to oxygen, raising the possibility that Wbl proteins might act as sensor proteins (17–21). To examine the *in vivo* requirement of WhiB for the [4Fe-4S] cluster, we carried out ChIP-seq analysis using a WhiB variant lacking the four conserved cysteine residues, thereby preventing cluster formation. *whiB* or FLAG-tagged *whiB* alleles encoding proteins in which the four cysteines were replaced with either four alanines (4C-A) or four serines (4C-S) were all unable to complement an *S. venezuelae whiB* mutant (see Fig. S7 in the supplemental material), similarly to observations made for each of the four single cysteine mutants of WhiD (17). Substitution of the four conserved cysteines did not reduce WhiB abundance or indirectly affect WhiA abundance (see Fig. S6). These results showed that the 4C-A and 4C-S variants of WhiB were phenotypically inactive, but to determine whether they were blocked in DNA binding or at a later stage, we carried out anti-FLAG ChIP-seq analysis of the 4C-S strain. The 4C-S 3×FLAG-WhiB protein was unable to bind to WhiAB target promoters *in vivo*, whereas high levels of enrichment of the same promoters were observed in the positive-control ChIP-seq experiment carried out in parallel on the equivalent strain expressing 3×FLAG-tagged wild-type WhiB (Fig. 6; see also Table S1D). We conclude that the [4Fe-4S] cluster of WhiB is essential for DNA binding *in vivo*. As expected, further ChIP-seq experiments showed that WhiA was unable to bind to the WhiAB target promoters in the 4C-S WhiB strain (data not shown).

### Conclusions.

**FIG 7:**
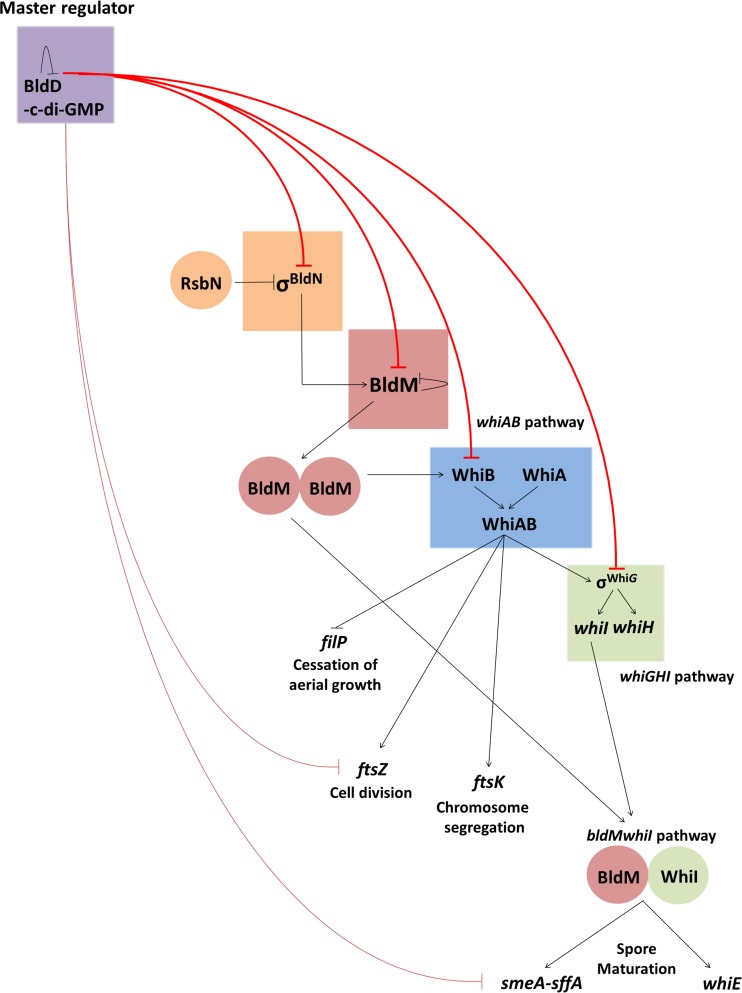
The regulatory network governing *Streptomyces* development. Flat-headed arrows indicate repression, and pointed arrows indicate activation. During vegetative growth, almost all of the genes of the core transcriptional regulatory cascade, including *bldN*, *bldM*, *whiB*, and *whiG* (upper red lines), are targets of BldD−(c-di-GMP)-mediated repression (3, 4, 47). BldD−(c-di-GMP) also represses genes encoding critical components of the cell division and chromosome segregation machineries required for sporulation septation, including FtsZ and SmeA-SffA (lower red lines). When the level of c-di-GMP perceived by BldD drops, BldD-mediated repression of almost the entire regulatory cascade is relieved. This allows σ^BldN^ to activate expression of BldM, which functions as a homodimer to activate expression of WhiB (33, 34). WhiAB proteins then coactivate key targets required for sporulation septation and chromosome segregation such as FtsZ, FtsW, and FtsK and the sporulation-specific sigma factor σ^WhiG^ that extends the regulatory cascade. σ^whiG^ directs expression of WhiI and WhiH (58, 59). Finally, WhiI forms a heterodimer with BldM to activate the expression of genes required for spore maturation (34), including the *smeA*-*sffA* operon involved in chromosome segregation into spores (60) and the multigene *whiE* locus that specifies the synthesis of the spore pigment (61).

The positions of WhiA and WhiB in the regulatory network governing *Streptomyces* development are illustrated in Fig. 7. During vegetative growth, the master regulator BldD−(c-di-GMP) complex represses expression of regulatory genes required both for formation of the aerial mycelium (e.g., *bldN* and *bldM*) and for subsequent sporulation in the aerial hyphae, such as *whiB* and *whiG* (3, 4, 47, 51). When the level of c-di-GMP perceived by BldD drops, BldD-mediated repression of almost the entire regulatory cascade is relieved, leading to derepression of *bldM* and of *whiB*. Subsequently, early in reproductive growth, BldM activates *whiB* expression (34). WhiAB proteins then coactivate the expression of key targets required for sporulation, including transcription factors that extend the regulatory cascade but also structural components of the cell division and chromosome segregation machineries that direct sporulation septation such as FtsZ, FtsW, and FtsK. The coactivation of expression of σ^WhiG^ by WhiAB establishes the first direct link between the *whiAB* pathway and the *whiGHI* pathway (3).

WhiA protein levels remain relatively constant throughout the *Streptomyces* life cycle (12). In contrast, ChIP-seq analysis shows that WhiA predominantly binds to the promoters of its target genes immediately prior to sporulation (12), strongly suggesting that WhiA activity is regulated posttranslationally. Given that WhiA and WhiB cocontrol the same regulon and their binding to the WhiAB target promoters is mutually dependent, it seems likely that WhiB is at least partially responsible for mediating this posttranslational control.

Our bioinformatic attempts to define a consensus binding sequence for WhiB using the *in vivo* targets found by ChIP-seq identified only the previously established WhiA consensus binding sequence, GACAC. Although our results show that WhiA and WhiB require each other to bind to their target promoters *in vivo*, we and others have already demonstrated that, *in vitro*, WhiA alone binds in a site-specific manner to DNA containing the sequence GACAC (11, 12). This sequence is highly abundant in the *S. venezuelae* genome (~15,000 copies, counting both strands), but ChIP-seq showed that only a small subset of these motifs is bound by WhiA *in vivo* (12), suggesting that there must be additional determinants of WhiA DNA binding. Our findings here raise the possibility that WhiB modulates the activity of WhiA to direct its binding to specific sequences *in vivo*.

Other members of the Wbl protein family have also been shown to require partner proteins for their function. For example, in *M. tuberculosis*, the function of the antibiotic resistance determinant WhiB7 (orthologous to *Streptomyces* WblC) as a transcriptional activator depends upon its interaction with the primary sigma factor SigA (52). Available evidence suggests that the C-terminal “AT-hook” of WhiB7 binds to *cis*-acting AT-rich elements found just upstream of the −35 promoter sequences recognized by SigA, acting to enhance the sigma factor specificity for distinct promoters. However, WhiB7/WblC is unique, and Wbl proteins (including WhiB) in general do not contain an AT-hook motif but rather a series of C-terminal basic residues. It seems likely that such motifs mediate weak binding to nonspecific DNA sequences, an observation that may explain why no clear consensus DNA-binding sequence has been established through studies of any Wbl protein, including the genome-wide analysis of the WhiB regulon presented here. We have shown that these C-terminal basic residues (WhiB 79 to 87) are essential for WhiB activity *in vivo* (data not shown), but we were unable to detect the WhiBΔ79–87–FLAG variant by Western blotting, raising the possibility that deletion of this region leads to WhiB instability. WhiB in *S. coelicolor* has been shown to bind to the *dpsA* promoter *in vitro* in EMSAs (30), but the *dpsA* gene is not conserved in *S. venezuelae*. To date we have been unable to demonstrate *in vitro* binding of *S. venezuelae* WhiB to any of the target promoters identified in this study. Indeed, we cannot formally rule out the possibility that WhiB interacts with DNA only via WhiA, although we consider this unlikely. In summary, we suggest that WhiB does not bind DNA specifically independently of WhiA but rather facilitates WhiA binding to DNA, acting as a discriminator to tailor WhiA selectivity *in vivo*. Given their cooperative function and close proximity *in vivo*, it seems likely that WhiA and WhiB must interact directly with each other.

## MATERIALS AND METHODS

### Bacterial strains, plasmids, oligonucleotides, and media.

Strains, plasmids, and oligonucleotides used in this study are described in Table S2 in the supplemental material. *Escherichia coli* K-12 strain DH5α was used for plasmid and cosmid propagation. BW25113 (53) containing a λ RED plasmid, pIJ790, was used to create disrupted cosmids. Cosmids and plasmids were conjugated from *dam dcm hsdS E. coli* strain ET12567 containing pUZ8002 (54) as described by Gust et al. (55, 56). *S. venezuelae* was grown either in liquid or on solid MYM (12). MYM (maltose 4 g l^−1^; Yeast extract 4 g l^−1^; malt extract 10 g l^−1^) was prepared using 50% tap water and 50% RO (reverse osmosis) water and after autoclaving was supplemented with 200 μl trace element solution per 100 ml.

### RNA isolation and DNA microarray analysis.

RNA isolation and DNA microarray analysis were performed as described previously (33, 57). The resulting data were processed to generate the graphs shown in this paper, according to a method described previously (12).

### Chromatin immunoprecipitation, library construction, sequencing, and ChIP-seq data analysis.

For the anti-FLAG experiments, ChIP was conducted using an M2 gel suspension (Sigma-Aldrich A2220) as described previously (12) for *S. venezuelae* strains ATCC 10712, SV7/pIJ10603 (*ΔwhiB*::*apr attB_ΦBT1_*::3×FLAG-[Gly_4_Ser]_3_-*whiB*), SV11/pIJ10601 (*ΔwhiA*::*apr attB_ΦBT1_*::3×FLAG-[Gly_4_Ser]_3_-*whiA*), SV7/pIJ10610 (*ΔwhiB*::*apr attB_ΦBT1_*::3×FLAG-[Gly_4_Ser]_3_-*whiB*[4C-S]), and SV51/pIJ10611 (*ΔwhiA ΔwhiB*::*apr attB_ΦBT1_*::3×FLAG-[Gly_4_Ser]_3_-*whiA*-*whiB*[4C-S]). For the anti-WhiB and anti-WhiA experiments, culture conditions were identical but ChIP was conducted using protein A-Sepharose (Sigma-Aldrich P3391) as described previously (47) for strains *S. venezuelae* ATCC 10712, SV11 (Δ*whiA*::*apr*), and SV7 (Δ*whiB*::*apr*). Library construction and sequencing were performed by The Genome Analysis Centre (TGAC), Norwich Research Park Norwich, United Kingdom, as described previously (12). ChIP-seq data analysis was conducted as described previously (12).

### Strain construction and complementation, Western blotting, and microscopy.

For details on strain construction and complementation, Western blotting, and microscopy, see Text S1 in the supplemental material (supplemental Materials and Methods).

## SUPPLEMENTAL MATERIAL

Figure S1  Complementation of the *whiA whiB* double mutant. Shown are the phenotypes of wild-type *S. venezuelae* (WT), the constructed *ΔwhiA ΔwhiB* null mutant SV51 (Δ*whiA* Δ*whiB*::*apr*), SV51 carrying the empty vector pMS82 (*ΔwhiA ΔwhiB*::*apr attB_ΦBT1_*::pMS82), and the complemented strains SV51-pIJ6760 (Δ*whiA* Δ*whiB*::*apr attB*_Φ*BT1*_::*whiA*), SV51-pIJ6761 (Δ*whiA* Δ*whiB*::*apr attB*_Φ*BT1*_::*whiB*), and SV51-pIJ10604 (Δ*whiA* Δ*whiB*::*apr attB*_Φ*BT1*_::*whiAB*). Sporulation is restored only when both *whiA* and *whiB* are expressed in *trans* from the ΦBT1 integration site. Strains were grown on MYM solid medium and photographed after 4 days. Download Figure S1, EPS file, 1.1 MB

Figure S2  WhiA and WhiB cannot promote sporulation independently of one another. (A) Phenotypes of the constructed *ΔwhiA* SV11 null mutant (Δ*whiA*::*apr*), the constructed Δ*whiB* SV7 null mutant (Δ*whiB*::*apr*), and the Δ*whiA* Δ*whiB* double mutant SV51 (Δ*whiA* Δ*whiB*::*apr*). The phenotype of each carrying the empty vector pIJ10257, *whiA* under ermE* control (pIJ10605), or *whiB* under ermE* control (pIJ10606) is shown. Strains were grown on MYM solid medium and photographed after 4 days. (B) Anti-WhiA Western blot confirming WhiA overexpression with *whiA* under ermE* control. WhiA was more abundant when overexpressed in the *whiA* (ΔwhiA ermE* *whiA*), *whiB* (ΔwhiB ermE* *whiA*), or *whiA whiB* (ΔwhiAB ermE* *whiA*) mutant background compared to the normal levels of WhiA detected in the wild-type strain (WT). WhiA was not detected in the negative control (*ΔwhiA*). Equal amounts (40 μg) of total protein were loaded from each sample. Download Figure S2, EPS file, 2.1 MB

Figure S3  WhiB ChIP-seq. (A) Construction of a functional FLAG-tagged version of WhiB. Shown are the phenotypes of wild-type *S. venezuelae* (WT), the constructed Δ*whiB*::*apr* SV7 null mutant (Δ*whiB*), SV7 carrying the empty vector pMS82 (Δ*whiB*::*apr attB_ΦBT1_*::pMS82), the complemented strain (Δ*whiB*::*apr attB*_Φ*BT1*_::*whiB*), and SV7 expressing an N-terminal, triple FLAG-tagged version of WhiB with an additional [Gly_4_Ser]_3_ linker (*ΔwhiB*::*apr attB_ΦBT1_*::3×FLAG-[Gly_4_Ser]_3_-*whiB*). Strains were grown on MYM solid medium and photographed after 4 days. (B) Chromosome-wide distribution of WhiB binding sites in *S. venezuelae* identified by ChIP-seq analysis. ChIP-seq analysis using M2 anti-FLAG antibody was conducted on the *ΔwhiB*::*apr attB_ΦBT1_*::3×FLAG-[Gly_4_Ser]_3_-*whiB* strain and on the wild-type strain (expressing nontagged WhiB from the native locus) as a negative control. ChIP-seq analysis using anti-WhiB (polyclonal) antibody was conducted on wild-type *S. venezuelae* (expressing WhiB from the native locus) and on the *ΔwhiB*::*apr* strain (lacking WhiB) as a negative control. All experiments were carried out at the onset of sporulation. Download Figure S3, EPS file, 1.9 MB

Figure S4  Comparison between anti-FLAG and anti-WhiB ChIP-seq data. ChIP traces are shown for 12 selected WhiB target genes: *sigN*, *infA*, *sven6396*/*wblH*, *cslA*, *pyrR*/*bldD*, *sven5479*/*nrdR*, *cvnA4*/*sven0992*, *sven1586*, *cvnA1*/*sven5239*, *sven5277*/*5278*, *sven5692*, and *sven2270*. Color coding of the ChIP samples is as follows: 3×FLAG-[Gly_4_Ser]_3_-WhiB anti-FLAG strain (WhiB anti-FLAG), red; *S. venezuelae* wild-type anti-FLAG negative control (WT anti-FLAG), purple; *S. venezuelae* wild-type anti-WhiB (WT anti-WhiB), green; *S. venezuelae* *ΔwhiB* anti-WhiB negative control (*ΔwhiB* anti-WhiB), blue. Plots span approximately 3 kb of DNA sequence. Genes running left to right are shown in green, and genes running right to left are shown in red. The black arrow indicates the gene(s) potentially subject to WhiB regulation. Download Figure S4, EPS file, 0.8 MB

Figure S5  WhiA is detected after WhiB ChIP. Data represent results of WhiA Western blot analysis (anti-WhiA polyclonal antibody; 1:2,500) following anti-FLAG ChIP (A) or anti-WhiB ChIP (B) in the strains indicated. WhiA is detected after anti-FLAG immunoprecipitation in the 3×FLAG-[Gly_4_Ser]_3_-WhiA strain (WhiA-FLAG, positive control) and in the 3×FLAG-[Gly_4_Ser]_3_-WhiB strain (WhiB-FLAG) but not in the wild-type strain (WT; negative control). WhiA was also detected after anti-WhiB immunoprecipitation in the wild-type strain (WT) and in the 3×FLAG-[Gly_4_Ser]_3_-WhiA strain (WhiA-FLAG) but not the Δ*whiB* SV7 strain (ΔB, negative control). The positions and sizes of WhiA (red arrow and red asterisks) and 3×FLAG-[Gly_4_Ser]_3_-WhiA (blue arrow and blue asterisks) are indicated against an NEB (no. P7706) protein ladder. Download Figure S5, EPS file, 0.4 MB

Figure S6  Western blot analysis. (A). Stabilities of FLAG-tagged versions of WhiB—with or without the [4Fe-4S] cluster and in the presence or absence of WhiA. Data represent results of Western blot analysis of WhiB (*ΔwhiB*::*apr attB_ΦBT1_*::3×FLAG-[Gly_4_Ser]_3_-*whiB*) (1), WhiB in the absence of *whiA* (*ΔwhiA ΔwhiB*::*apr attB_ΦBT1_*::3×FLAG-[Gly_4_Ser]_3_-*whiB*) (2), the WhiB cysteine-serine variant (*ΔwhiB*::*apr attB_ΦBT1_*::3×FLAG-[Gly_4_Ser]_3_-*whiB* [4C-S]) (3), the WhiB cysteine-serine variant in the absence of *whiA* (*ΔwhiA ΔwhiB*::*apr attB_ΦBT1_*::3×FLAG-[Gly_4_Ser]_3_-*whiB* [4C-S]) (4), and wild-type *S. venezuelae* (5). The position of 3×FLAG-[Gly_4_Ser]_3_-WhiB is indicated by a red arrow; 3×FLAG-[Gly_4_Ser]_3_-WhiB was absent in the negative control (wild type; 5). Equal amounts (30 µg) of total protein were loaded from each sample. (B) Stability of FLAG-tagged WhiA in the absence of WhiB and in the presence of WhiB without the [4Fe-4S] cluster. Data represent results of Western blot analysis of WhiA (Δ*whiA*::*apr attB_ΦBT1_*::3×FLAG-[Gly_4_Ser]_3_-*whiA*) (1), WhiA in the absence of *whiB* (*ΔwhiA ΔwhiB*::*apr attB_ΦBT1_*::3×FLAG-[Gly_4_Ser]_3_-*whiA*) (2), WhiA in the presence of the WhiB cysteine-serine variant (*ΔwhiA ΔwhiB*::*apr attB_ΦBT1_*::3×FLAG-[Gly_4_Ser]_3_-*whiA*::*whiB* [4C-S]) (3), and wild-type *S. venezuelae* (4). The position of 3×FLAG-[Gly_4_Ser]_3_-WhiA is indicated by a red arrow; 3×FLAG-[Gly_4_Ser]_3_-WhiA was absent in the negative control (wild type; 4). Equal amounts (40 µg) of total protein were loaded from each sample. Download Figure S6, EPS file, 1.7 MB

Figure S7  Cysteine variants of WhiB are unable to promote sporulation. (A) Comparison of the phenotypes of wild-type *S. venezuelae* (WT), the *ΔwhiB* SV7 null mutant (Δ*whiB*::*apr*), SV7 carrying the empty vector pMS82 (*ΔwhiB*::*apr attB_ΦBT1_*::pMS82), the complemented strain (Δ*whiB*::*apr attB*_Φ*BT1*_::*whiB*), the WhiB Cys–Ala variant complemented strain (*ΔwhiB*::*apr attB_ΦBT1_*::*whiB*[4C-A]), and the WhiB Cys–Ser variant complemented strain (*ΔwhiB*::*apr attB_ΦBT1_*::*whiB*[4C-S]). (B) Comparison of the phenotypes of wild-type *S. venezuelae* (WT), the Δ*whiB* SV7 null mutant (Δ*whiB*::*apr*), SV7 carrying the empty vector pMS82 (*ΔwhiB*::*apr attB_ΦBT1_*::pMS82), the FLAG complemented strain (Δ*whiB*::*apr attB*_Φ*BT1*_::3×FLAG-*whiB*), the WhiB Cys–Ala FLAG variant complemented strain (*ΔwhiB*::*apr attB_ΦBT1_*::3×FLAG-*whiB*[4C-A]), and the WhiB Cys–Ser FLAG variant complemented strain (*ΔwhiB*::*apr attB_ΦBT1_*::3×FLAG-*whiB*[4C-S]). Strains were grown on MYM solid medium and photographed after 4 days. Download Figure S7, EPS file, 1.4 MB

Table S1  (A) Complete ChIP-seq data set for *S. venezuelae* WT (anti-WhiB) and the *ΔwhiB*::*apr attB_ΦBT1_*::3×FLAG-[Gly_4_Ser]_3-_*whiB* (anti-FLAG) strain. Each row represents a ChIP “peak” based on the analysis of 25-bp segments of the *S. venezuelae* genome. Only those peaks with significance at a *P* value of <E-04 for at least one of the ChIP samples are included in the analysis. Pos, position of peak in the *S. venezuelae* genome in bases; diff, the difference between the local normalized (ln) values of the immunoprecipitated (ChIP) samples and the total (non-ChIP) DNA samples for each of the ChIP samples, i.e., the Δ*whiB*::FLAG-*whiB*/FLAG (anti-FLAG sample), WT/FLAG (anti-FLAG negative control), WT/polyclonal (POLY) (anti-WhiB sample), and Δ*whiB*/POLY (anti-WhiB negative control) strains. Adjusted *P* values (apv), significance values for each of the ChIP samples after adjusting for multiple testing by the Hochberg method as implemented in the *p.adjust* function of (R). Peak (pk), each ChIP sample was qualified as being significant (*P* < E-04) by the identifier “TRUE” and nonsignificant (*P* > E-04) by the identifier “FALSE.” Left Gene, the identifier (SVEN no.) for the gene on the left of the identified ChIP peak. Right Gene, the identifier (SVEN no.) for the gene on the right of the identified ChIP peak. Distance, the distance (in bases) between the ChIP peak and the predicted start codon of the downstream gene. Start, the start position on the *S. venezuelae* genome (in bases) of the gene downstream of the ChIP peak. End, the end position on the *S. venezuelae* genome (in bases) of the gene downstream of the ChIP peak. Strand, the strand on which the gene is found (forward = 1; reverse = −1). Product, (possible) gene function based on annotation in StrepDB (http://strepdb.streptomyces.org.uk). Affy LogFC, the log-fold change (log_2_ scale) in expression of the *whiB* mutant SV7 compared to wild-type *S. venezuelae* at the 10-, 12-, 14-, 16-, 18-, and 20-h time points. (−), decrease in expression of the gene in a *whiB* mutant compared to the wild-type strain; (+), increase in expression of the gene in a *whiB* mutant compared to the wild-type strain. Cells highlighted in red represent greater than a 2-fold increase in expression in the *whiB* mutant. Cells highlighted in yellow represent greater than a 2-fold decrease in expression in the *whiB* mutant. (B) ChIP-seq data set for the *S. venezuelae* WT (anti-WhiB) and *ΔwhiB*::*apr attB_ΦBT1_*::3×FLAG-[Gly_4_Ser]_3-_*whiB* (anti-FLAG) strains for significant positions present in the “WT/FLAG” or “Δ*whiB*/POLY” negative control that are also associated with significant peaks in the “Δ*whiB*::FLAG-*whiB*/FLAG” or “WT/POLY” ChIP sample. Each row represents a ChIP “peak” based on the analysis of 25-bp segments of the *S. venezuelae* genome. Only those peaks with significance values of *P* of <E-04 for at least one of the ChIP samples are included in the analysis. Pos, position of peak in the *S. venezuelae* genome in bases. diff, the difference between the local normalized (ln) values of the immunoprecipitated (ChIP) samples and the total (non-ChIP) DNA samples, for each of the ChIP samples, i.e., Δ*whiB*::FLAG-*whiB*/FLAG (anti-FLAG sample), WT/FLAG (anti-FLAG negative control), WT/POLY (anti-WhiB sample), Δ*whiB*/POLY (anti-WhiB negative control). Adjusted *P* values (apv), significance values for each of the ChIP samples after adjusting for multiple testing by the Hochberg method as implemented in the *p.adjust* function of (R). Peak (pk), each ChIP sample is qualified as being significant (*P* < E-04) by the identifier “TRUE” and nonsignificant (*P* > E-04) by the identifier “FALSE.” Left Gene, the identifier (SVEN no.) for the gene on the left of the identified ChIP peak. Right Gene, the identifier (SVEN no.) for the gene on the right of the identified ChIP peak. Start, the start position on the *S. venezuelae* genome (in bases) of the gene downstream of the ChIP peak. End, the end position on the *S. venezuelae* genome (in bases) of the gene downstream of the ChIP peak. Strand, the strand on which the gene is found (forward = 1; reverse = −1). Product, (possible) gene function based on annotation in StrepDB (http://strepdb.streptomyces.org.uk). (C) ChIP-seq data set for *S. venezuelae* WT (anti-WhiB) in the presence or absence of *whiA* and for *S. venezuelae* WT (anti-WhiA) in the presence or absence of *whiB.* Each row represents a ChIP “peak” based on the analysis of 25-bp segments of the *S. venezuelae* genome. Only those peaks with significance values of *P* of <E-04 for at least one of the ChIP samples are included in the analysis. Pos, position of peak in the *S. venezuelae* genome in bases. diff, the difference between the local normalized (ln) values of the immunoprecipitated (ChIP) samples and the total (non-ChIP) DNA samples, for each of the ChIP samples, i.e., the WT/anti-WhiB strain (anti-WhiB sample in the presence of *whiA*), the Δ*whiB*/anti-WhiB strain (anti-WhiB negative control), the Δ*whiA*/anti-WhiB strain (anti-WhiB sample in the absence of *whiA*), the WT/anti-WhiA strain (anti-WhiA sample in the presence of *whiB*), the Δ*whiA*/anti-WhiA strain (anti-WhiA negative control), and the Δ*whiB*/anti-WhiA strain (anti-WhiA sample in the absence of *whiB*). Adjusted *P* values (apv), significance values for each of the ChIP samples after adjusting for multiple testing by the Hochberg method as implemented in the *p.adjust* function of (R). Peak (pk), each ChIP sample is qualified as being significant (*P* < E-04) by the identifier “TRUE” and nonsignificant (*P* > E-04) by the identifier “FALSE.” Left Gene, the identifier (SVEN no.) for the gene on the left of the identified ChIP peak. Right Gene, the identifier (SVEN no.) for the gene on the right of the identified ChIP peak. Start, the start position on the *S. venezuelae* genome (in bases) of the gene downstream of the ChIP peak. End, the end position on the *S. venezuelae* genome (in bases) of the gene downstream of the ChIP peak. Strand, the strand on which the gene is found (forward = 1; reverse = −1). Product, (possible) gene function based on annotation in StrepDB (http://strepdb.streptomyces.org.uk). The WhiA polyclonal antibody appears to cross-react with other DNA-associated proteins. However, the WhiA-dependent significant positions are dependent upon both *whiA* and *whiB*. (D) Complete ChIP-seq data set for the *S. venezuelae* Δ*whiB*::*apr attB_ΦBT1_*::3×FLAG-[Gly_4_Ser]_3-_*whiB* and Δ*whiB*::*apr attB_ΦBT1_*::3×FLAG-[Gly_4_Ser]_3-_*whiB*(4C-S) (anti-FLAG) strains. Each row represents a ChIP “peak” based on the analysis of 25-bp segments of the *S. venezuelae* genome. Only those peaks with significance values of *P* of <E-04 for at least one of the ChIP samples are included in the analysis. Pos, position of peak in the *S. venezuelae* genome in bases. diff, the difference between the local normalized (ln) values of the immunoprecipitated (ChIP) samples and the total (non-ChIP) DNA samples, for each of the ChIP samples, i.e., the Δ*whiB*::FLAG-*whiB*/FLAG strain (anti-FLAG sample), the Δ*whiB*::FLAG-*whiB*(4C-S)/FLAG strain (anti-FLAG sample), and the WT/FLAG strain (anti-FLAG negative control). Adjusted *P* values (apv), significance values for each of the ChIP samples after adjusting for multiple testing by the Hochberg method as implemented in the *p.adjust* function of (R). Peak (pk), each ChIP sample is qualified as being significant (*P* < E-04) by the identifier “TRUE” and nonsignificant (*P* > E-04) by the identifier “FALSE.” Left Gene, the identifier (SVEN no.) for the gene on the left of the identified ChIP peak. Right Gene, the identifier (SVEN no.) for the gene on the right of the identified ChIP peak. Distance, the distance (in bases) between the ChIP peak and the predicted start codon of the downstream gene. Start, the start position on the *S. venezuelae* genome (in bases) of the gene downstream of the ChIP peak. End, the end position on the *S. venezuelae* genome (in bases) of the gene downstream of the ChIP peak. Strand, the strand on which the gene is found (forward = 1; reverse = −1). Product, (possible) gene function based on annotation in StrepDB (http://strepdb.streptomyces.org.uk). Affy LogFC, the log-fold change (log_2_ scale) in expression of the *whiB* mutant SV7 compared to wild-type *S. venezuelae* at the 10-, 12-, 14-, 16-, 18-, and 20-h time points. (−), decrease in expression of the gene in a *whiB* mutant compared to the wild-type strain; (+), increase in expression of the gene in a *whiB* mutant compared to the wild-type strain. Cells highlighted in red represent greater than a 2-fold increase in expression in the *whiB* mutant. Cells highlighted in yellow represent greater than a 2-fold decrease in expression in the *whiB* mutant. Table S1, XLSX file, 0.4 MB

Table S2  Strains, plasmids, and oligonucleotide primers used in this study. Table S2, DOCX file, 0.03 MB

Text S1  Supplemental Materials and Methods. Download Text S1, DOCX file, 0.03 MB
