# Supplementary material for: Genome-Wide Chromatin Immunoprecipitation Sequencing Analysis Shows that WhiB Is a Transcription Factor That Cocontrols Its Regulon with WhiA To Initiate Developmental Cell Division in Streptomyces
Source: mBio. 2016 Apr 19;7(2):e00523-16. doi: 10.1128/mBio.00523-16 (PMC4850268; doi:10.1128/mBio.00523-16)
Supplement: Table S2 — Strains, plasmids, and oligonucleotide primers used in this study. [file mbo002162789st2.docx]

Table S2 Strains, Plasmids and Oligonucleotide primers used in this study

**Strains Relevant genotype/comments Source/reference**

*S.venezuelae*

ATCC10712 Wild-type

SV11 Δ*whiA::apr* (1)

SV11/pIJ6760 Δ*whiA::apr* with pIJ6760 integrated at the ΦBT1 attachment site (1)

SV11/pIJ10601 Δ*whiA::apr* with pIJ10601 integrated at the ΦBT1 attachment site (1)

SV7 Δ*whiB::apr* This work

SV7/pIJ6761 Δ*whiB::apr* with pIJ6761 integrated at the ΦBT1 attachment site This work

SV7/pIJ10602 Δ*whiB::apr* with pIJ10602 integrated at the ΦBT1 attachment site This work

SV7/pIJ10603 Δ*whiB::apr* with pIJ10603 integrated at the ΦBT1 attachment site This work

SV50 Δ*whiA* This work

SV50/pIJ6760 Δ*whiA* with pIJ6760 integrated at the ΦBT1 attachment site This work

SV51 Δ*whiA* Δ*whiB::apr* This work

SV51/pIJ10601 Δ*whiA* Δ*whiB::apr* with pIJ10601 integrated at the ΦBT1 attachment site This work

SV51/pIJ10603 Δ*whiA* Δ*whiB::apr* with pIJ10603 integrated at the ΦBT1 attachment site This work

SV51/pIJ10604 Δ*whiA* Δ*whiB::apr* with pIJ10604 integrated at the ΦBT1 attachment site This work

SV51/pIJ6760 Δ*whiA* Δ*whiB::apr* with pIJ6760 integrated at the ΦBT1 attachment site This work

SV51/pIJ6761 Δ*whiA* Δ*whiB::apr* with pIJ6761 integrated at the ΦBT1 attachment site This work

SV11/pIJ10605 Δ*whiA::apr* with pIJ10605 integrated at the ΦBT1 attachment site This work

SV11/pIJ10606 Δ*whiA::apr* with pIJ10606 integrated at the ΦBT1 attachment site This work

SV7/pIJ10605 Δ*whiB::apr* with pIJ10605 integrated at the ΦBT1 attachment site This work

SV7/pIJ10606 Δ*whiB::apr* with pIJ10606 integrated at the ΦBT1 attachment site This work

SV7/pIJ10607 Δ*whiB::apr* with pIJ10607 integrated at the ΦBT1 attachment site This work

SV7/pIJ10608 Δ*whiB::apr* with pIJ10608 integrated at the ΦBT1 attachment site This work

SV7/pIJ10609 Δ*whiB::apr* with pIJ10609 integrated at the ΦBT1 attachment site This work

SV7/pIJ10610 Δ*whiB::apr* with pIJ10610 integrated at the ΦBT1 attachment site This work

SV51/pIJ10610 Δ*whiA* Δ*whiB::apr* with pIJ10610 integrated at the ΦBT1 attachment site This work

SV51/pIJ10611 Δ*whiA* Δ*whiB::apr* with pIJ10611 integrated at the ΦBT1 attachment site This work

SV51/pIJ10257 Δ*whiA* Δ*whiB::apr* with pIJ10257 integrated at the ΦBT1 attachment site This work

SV51/pIJ10605 Δ*whiA* Δ*whiB::apr* with pIJ10605 integrated at the ΦBT1 attachment site This work

SV51/pIJ10606 Δ*whiA* Δ*whiB::apr* with pIJ10606 integrated at the ΦBT1 attachment site This work

*E.coli*

ET12567(pUZ8002) ET12567 containing helper plasmid pUZ8002 (2)

BW25113 Δ(*araD*-*araB*)*567* Δ *lacZ4787*(::*rrnB-4*) *lacIp-4000*(*lacI^Q^*), l*-rpoS369*(*Am*) *rph-1* (3)

Δ(*rhaD*-*rhaB*)*568 hsdR514*

**Plasmids**

pIJ773 Plasmid template for amplification of the *apr oriT* cassette for ‘Redirect’ PCR-targeting (4)

pIJ790 Modified λ RED recombination plasmid [*oriR101*] [*repA101*(ts)] *araBp-gam-bet-exo* (4)

pMS82 Plasmid cloning vector for the conjugal transfer of DNA from *E. coli* to *Streptomyces* spp. (5)

Integrates site specifically at the ΦBT1 attachment site (Hyg^R^)

pIJ10257 Plasmid cloning vector for the conjugal transfer of DNA (under control of the ermE* constitutive (6)

promoter) from *E. coli* to *Streptomyces* spp. Integrates site specifically at the ΦBT1 attachment

site (Hyg^R^).

pIJ12738 Delivery vector (containing the I-SceI recognition site) required for I-SceI-mediated gene (7)

deletions

pIJ2742 Temperature sensitive plasmid (pGM1190-based) encoding the I-SceI Meganuclease (7)

pIJ6760 pMS82 carrying *whiA* driven from its own promoter (1)

pIJ10601 pMS82 carrying sequence encoding 3xFLAG-[Gly_4_Ser]_3_-WhiA driven from *whiA* promoter (1)

pIJ6761 pMS82 carrying *whiB* driven from its own promoter This work

pIJ10602 pMS82 carrying sequence encoding 3xFLAG-WhiB driven from its own promoter This work

pIJ10603 pMS82 carrying sequence encoding 3xFLAG-[Gly_4_Ser]_3_-WhiB driven from *whiB* promoter This work

pIJ10750 pMS82 with an extended Multiple Cloning Site (MCS) This work

pIJ10604 pIJ10750 carrying *whiA* and *whiB* driven from their own promoters This work

pIJ10605 pIJ10257 carrying *whiA*, driven by ermE* This work

pIJ10606 pIJ10257 carrying *whiB*, driven by ermE* This work

pIJ10607 pMS82 carrying a version of *whiB* encoding WhiB (C25A, C48A, C51A and C57A) This work

driven by its own promoter

pIJ10608 pMS82 carrying a version of *whiB* encoding WhiB (C25S, C48S, C51S and C57S) This work

driven by its own promoter

pIJ10609 pMS82 carrying a version of *3xFLAG-[Gly_4_Ser]_3_-whiB* encoding 3xFLAG-[Gly_4_Ser]_3_-WhiB This work

(C25A, C48A, C51A and C57A) driven by *whiB* promoter

pIJ10610 pMS82 carrying a version of *3xFLAG-[Gly_4_Ser]_3_-whiB* encoding 3xFLAG-[Gly_4_Ser]_3_-WhiB This work

(C25S, C48S, C51S and C57S) driven by *whiB* promoter

pIJ10611 pIJ10750 carrying sequence encoding 3xFLAG-[Gly_4_Ser]_3_-WhiA driven from *whiA* promoter This work

and a sequence encoding WhiB (C25S, C48S, C51S and C57S) driven by *whiB* promoter

**Primers** **Sequence**

whiBdis_F caccacggggacgcacagaacagacgaggggcgcacatgattccggggatccgtcgacc

whiBdis_R ggcctggcgcgcggggcccggcaggcttcgcgccgttcatgtaggctggagctgcttc

whiBcon_F cagggcggggggacgtcggg

whiBcon_R gcggggtcctgatcggtccg

whiBcomp_F cggaagcttgccggtggcatgtgtcac

whiBcomp_R cggaagcttgcaggcttcgcgccgttc

whiAISceI_FLANKA_F ggcgaagcttgaacacggcgacaggagag

whiAISceI_FLANKA_R cctccgatatccatcgccatgggccgatc

whiAISceI_FLANKB_F cctccgatatctgaccggagccgtcccc

whiAISceI_FLANKB_R ggggtaccgctcggcgccggtgtacca

whiAext_F gacgtccagtccgtgcac

whiAext_R gggctgaagcaggagatcc

whiAcomp_82MCS_F tccgctcgagcggagaaaccgagggcttctc

whiAcomp_82MCS_R cggggtacctccggggacggctccggtc

whiBcomp_82MCS_F gggaattccatatggccggtggcatgtgtcac

whiBcomp_82MCS_R gctgcctagggcaggcttcgcgccgttc

whiBFLAGext_F ggtccgtgaccggactgt

whiBFLAGfus_R tcgatgtcgtggtccttgtagtcgccgtcgtggtccttgtagtccatgtgcgcccctcgtctgtt

whiBFLAGfus_F cgactacaaggaccacgacatcgactacaaggacgatgacgacaagatgaccgagttgttccagg

whiBFLAGext_R gagcgtctcggacggag

whiBFLAGnes_F ggcgaagcttgccggtggcatgtgtcac

whiBFLAGnes_R ggggtaccgcaggcttcgcgccgttc

whiBLINKfus_F gttcaggcggaggtggctctggcggtggcggtagtatgaccgagttgttccaggaa

whiBLINKfus_R cgccagagccacctccgcctgaaccgcctccacccttgtcgtcatcgtccttgtag

Mcs-1Fwd ctagaaagcttagattctctcatatgatcatcgattcgcgacttaagcctaggtgtacatctctcgagttaattaaggtac

Mcs-1Rev cttaattaactcgagagatgtacacctaggcttaagtcgcgaatcgatgatcatatgagagaatctaagcttt

whiApIJ10257_F gggaattccatatggcgatgacggcagcg

whiApIJ10257_R cccaagctttcagccgaccaggctgtc

whiBpIJ10257_F gggaattccatatgaccgagttgttccagga

whiBpIJ10257_R cccaagctttcagacggcggccttctt

**REFERENCES**

1. [**Bush MJ**](http://www.ncbi.nlm.nih.gov/pubmed/?term=Bush%20MJ%5BAuthor%5D&cauthor=true&cauthor_uid=24065632)**,** [**Bibb MJ**](http://www.ncbi.nlm.nih.gov/pubmed/?term=Bibb%20MJ%5BAuthor%5D&cauthor=true&cauthor_uid=24065632)**,** [**Chandra G**](http://www.ncbi.nlm.nih.gov/pubmed/?term=Chandra%20G%5BAuthor%5D&cauthor=true&cauthor_uid=24065632)**,** [**Findlay KC**](http://www.ncbi.nlm.nih.gov/pubmed/?term=Findlay%20KC%5BAuthor%5D&cauthor=true&cauthor_uid=24065632)**,** [**Buttner MJ**](http://www.ncbi.nlm.nih.gov/pubmed/?term=Buttner%20MJ%5BAuthor%5D&cauthor=true&cauthor_uid=24065632). 2013. Genes required for aerial growth, cell division, and chromosome segregation are targets of WhiA before sporulation in *Streptomyces venezuelae*. [MBio.](http://www.ncbi.nlm.nih.gov/pubmed/?term=bush+whia) **4:**e00684–13.
2. **Paget MSB, Chamberlin L, Atrih A, Foster SJ, Buttner MJ**. 1999. Evidence that the extracytoplasmic function sigma factor, σ^E^, is required for normal cell wall structure in *Streptomyces coelicolor* A3(2). J. Bacteriol. **181:**204–211.
3. **Datsenko KA, Wanner BL**. 2000. One-step inactivation of chromosomal genes in *Escherichia coli* K-12 using PCR products. Proc. Natl. Acad. Sci. U. S. A. **97:**6640–6645.
4. **Gust B, Challis GL, Fowler K, Kieser T, Chater KF**. 2003. PCR-targeted *Streptomyces* gene replacement identifies a protein domain needed for biosynthesis of the sesquiterpene soil odor geosmin. Proc. Natl. Acad. Sci. U. S. A. **100:**1541–1546.
5. **Gregory MA, Till R, Smith MCM**. 2003. Integration site for *Streptomyces* phage ΦBT1 and development of site-specific integrating vectors. J. Bacteriol. **185:**5320–5323.
6. **Hong HJ, Hutchings MI, Hill LM, Buttner MJ**. 2005. [The role of the novel Fem protein VanK in vancomycin resistance in Streptomyces coelicolor.](http://www.ncbi.nlm.nih.gov/pubmed/15632111) J Biol Chem. **280**:13055-61.
7. [**Fernández-Martínez LT**](http://www.ncbi.nlm.nih.gov/pubmed/?term=Fern%C3%A1ndez-Mart%C3%ADnez%20LT%5BAuthor%5D&cauthor=true&cauthor_uid=25403842)**,** [**Bibb MJ**](http://www.ncbi.nlm.nih.gov/pubmed/?term=Bibb%20MJ%5BAuthor%5D&cauthor=true&cauthor_uid=25403842). 2014 Use of the meganuclease I-SceI of *Saccharomyces cerevisiae* to select for gene deletions in actinomycetes. [Sci Rep.](http://www.ncbi.nlm.nih.gov/pubmed/?term=iscei+bibb) **4:**7100.
