# Supplementary material for: Genome-Wide Chromatin Immunoprecipitation Sequencing Analysis Shows that WhiB Is a Transcription Factor That Cocontrols Its Regulon with WhiA To Initiate Developmental Cell Division in Streptomyces
Source: mBio. 2016 Apr 19;7(2):e00523-16. doi: 10.1128/mBio.00523-16 (PMC4850268; doi:10.1128/mBio.00523-16)
Supplement: Text S1 — Supplemental Materials and Methods. Download [file mbo002162789s1.docx]

**Construction and complementation of *S. venezuelae whiB* null mutant.** Using the ‘Redirect’ PCR targeting method of Gust *et al*. (1, 2), *whiB* mutants were generated in which the coding region was precisely replaced by an apramycin resistance (*apr*) cassette. A cosmid library that covers > 98% of the *S. venezuelae* genome (M.J. Bibb and M.J. Buttner, unpublished) is fully documented at http://strepdb.streptomyces.org.uk/. Cosmid 1C3 was introduced into *E. coli* BW25113 containing pIJ790 and the *whiB* gene was replaced with the *apr-oriT* cassette amplified from pIJ773 using the primer pair whiBdis_F and whiBdis_R. The resulting disrupted cosmid was confirmed by restriction digestion and by PCR analysis using the flanking primers whiBcon_F and whiBcon_R, and introduced into *S. venezuelae* by conjugation. Null mutant derivatives, generated by double crossing over, were identified by their apramycin-resistant, kanamycin-sensitive and morphological phenotypes, and their chromosomal structures were confirmed by PCR analysis using the flanking primers whiBcon_F and whiBcon_R and by Southern hybridization using the entire cosmid 1C3, partially digested with Sau3A1, as a probe. A representative *whiB* null mutant was designated SV7. For complementation, *whiB* was amplified with the primers whiBcomp_F and whiBcomp_R, generating a fragment carrying the coding sequence and the *whiB* promoter, and cloned into HindIII-cut pMS82 (3) to create pIJ6761. The plasmid was introduced into the *whiB* mutant by conjugation and fully complemented all aspects of the mutant phenotype.

**I-SceI Meganuclease-mediated gene deletion.** Using the I-SceI Meganuclease method of gene deletion (4), a markerless *ΔwhiA* mutant was generated. Approximately ~2kb sequences upstream and downstream of the *whiA* gene were first cloned into the pIJ12738 delivery vector (that contains the I-SceI recognition site) using the whiAISceI_FLANKA_F/R and whiAISceI_FLANKB_F/R primer pairs and the HindIII/EcoRV and the EcoRV/KpnI restriction enzymes, respectively. The resulting construct was introduced into wild-type *S. venezuelae* by conjugation and single cross-overs selected for on the basis of apramycin resistance. This was confirmed by colony PCR using the primers *whiA*ext_F and *whiA*ext_R and spore preparations of the strain carried out. A plasmid encoding the I-SceI Meganuclease (pIJ2742) was then conjugated into the single cross-over strain and selected for on the basis of thiostrepton resistance. Ex-conjugants were grown on media containing 50 µg/ml thiostrepton and double cross-overs counter-selected on the basis of apramycin sensitivity and morphological phenotypes before confirmation using the primers *whiA*ext_F and *whiA*ext_R. As a derivative of pGM1190 (5), pIJ2742 has a temperature sensitive origin of replication. Selected *whiA* mutants were therefore grown at 37ºC, to promoter loss of the plasmid. A representative *whiA* mutant was designated SV50. For complementation, the pIJ6760 plasmid (6), carrying the *whiA* gene under control of its native promoter was introduced into the *whiA* mutant and fully complemented all aspects of the mutant phenotype.

**SV1 phage transduction.** The marked *ΔwhiB::apr* allele (from SV7) was moved into the *ΔwhiA* strain SV50 by generalised transduction using the *S. venezuelae*-specific phage SV1 (7). To prepare SV1-lysate, 20 µl volumes from a range of SV1 dilutions (e.g. 10^0^-10^-8^) was added to 15 µl volumes of mycelial fragments, prepared from the *ΔwhiB::apr* donor strain SV7 and mixed in 800 μl pre-warmed (45°C) soft nutrient agar (SNA) before pouring onto Difco nutrient agar plates containing 0.5% glucose, 10 mM MgSO_4_ and 10 mM Ca(NO_3_)_2_. The plates were incubated at 30°C overnight, then near-confluent plates flooded with 2.5 ml Difco nutrient broth (DNB) and incubated for 3-4 hr at room temperature. The phage-containing DNB soak-out was harvested and filtered through a 0.45 μm filter to eliminate bacterial contamination. For transduction of the *Δ*whiB::apr allele, 100 µl of phage soak-out harvested from the *Δ*whiB::apr mutant strain SV7, was mixed with 10 µl of mycelial fragments prepared from the *Δ*whiA SV50 strain and incubated overnight on MYM agar at room temperature before overlaying with apramycin for selection. Plates spread with the recipient strain or the phage alone were used as controls. Transduction of the *Δ*whiB::apr allele was confirmed by PCR using the whiBcon_F and whiBcon_R primers and a representative *ΔwhiA* *ΔwhiB::apr* double mutant was named SV51.

**Complementation of the *whiA whiB* double mutant.** For complementation, the *whiA* and *whiB* genes were cloned in tandem into a modified form of pMS82 containing an extended multiple cloning site (MCS). This version of pMS82 (pIJ10750) was created using phosphorylated Mcs1_Fwd and Mcs1_Rev primers that were annealed before ligation between the AvrII and KpnI sites of pMS82, thereby extending the MCS and providing additional and unique NdeI, ClaI, NruI, AflII, BsrGI, XhoI and PacI restriction sites, with the loss of the NsiI site. For *whiA*, the gene under control of its native promoter was cloned using the whiAcomp_82MCS_F/R primer pair via the XhoI and KpnI restriction sites respectively. For *whiB,* the gene under control of its native promoter was cloned using the whiBcomp_82MCS_F/R primer pair via the NdeI and AvrII restriction sites respectively. This generated pIJ10604 which was introduced into the ∆*whiA* ∆*whiB::apr* strain SV51 by conjugation and fully complemented all aspects of the mutant phenotype.

**Construction of *whiA* and *whiB* overexpression strains.** For overexpression of *whiA*, the *whiA* coding region was amplified using the whiApIJ10257_F and whiApIJ10257_R primers. For overexpression of *whiB*, the *whiB* coding region was amplified using the whiBpIJ10257_F and whiBpIJ10257_R primers. Both *whiA* and *whiB* Forward and Reverse primers contain flanking NdeI and HindIII sites respectively, which were used to clone the two PCR fragments into NdeI/HindIII-cut pIJ10257 to create pIJ10605 and pIJ10606 respectively. pIJ10605 and pIJ10606 were introduced into the SV11 (*ΔwhiA::apr*), SV7 (*ΔwhiB::apr*) and SV51 (*ΔwhiA ΔwhiB::apr*) strains by conjugation and the ability of the strains to sporulate on MYM was assessed.

**Construction of the 3xFLAG-WhiB complemented *S. venezuelae* strain.** To engineer an *S. venezuelae* strain expressing WhiB with an N-terminal, triple-FLAG tag (MDYKDHDGDYKDHDIDYKDDDDK), a pMS82-derived construct, pIJ10602, was created via a two-step fusion-PCR approach. In the first step, the 1C3 cosmid was used as a template for two separate PCR-reactions. The first reaction amplified the promoter region of the *whiB* gene using the primer pair whiBFLAGext_F and whiBFLAGfus_R. The second reaction amplified the coding region of the *whiB* gene using the primer pair whiBFLAGfus_F and whiBFLAGext_R. Together the whiBFLAGfus_R and whiBFLAGfus_F primers contain the sequence encoding the triple-FLAG tag via a 24bp overlapping section. In the second step, a PCR reaction using the nested primers whiBFLAGnes_F and whiBFLAGnes_R was used to amplify the entire *whiB* gene and its promoter, fusing the two products from step 1 together and incorporating the 3xFLAG tag sequence between them. The nested primers whiBFLAGnesfor and WhiBFLAGnesrev additionally contain the HindIII and KpnI sites respectively to enable cloning into HindIII, KpnI-cut pMS82. The resulting vector was named pIJ10602. In order to insert a [Gly_4_Ser]_3_ linker between the 3xFLAG tag and the coding region of *whiB*, a second fusion experiment was conducted using pIJ10602 as a template. Once again two PCR reactions were carried out in a first step using the primer pairs whiBFLAGnes_F/whiBLINKfus_R and WhiBFLAGnes_R/whiBLINKfus_F. Together the whiBLINKfus_R and whiBLINKfus_R primers contain the sequence encoding the [Gly_4_Ser]_3_ linker (ggtggaggcggttcaggcggaggtggctctggcggtggcggtagt) via a 24 bp overlapping section. The second step, employing the same nested primers resulted in fusion of the two products in the first step and incorporated the linker sequence between them. Restriction digestion using HindIII and KpnI, followed by ligation into HindIII, KpnI-cut pMS82, created pIJ10603. The plasmid pIJ10603 was introduced into the *ΔwhiB::apr* mutant SV7 by conjugation and its ability to restore sporulation was assessed both on solid and in liquid MYM medium.

**Construction of WhiB-variant strains.** Variants of WhiB lacking all four conserved cysteines (C25, C48, C51 and C57) were created. Mutant alleles of *whiB* and *3xFLAG-[Gly_4_Ser]_3_-whiB* were synthesized in which all four cysteine TGC codons were altered either to alanine GGC codons or serine TGC codons (GenScript). These alleles were subcloned into pMS82 using the primer pair whiBFLAGnes_F/R to create the plasmids pIJ10607, pIJ10608, pIJ10609 and pIJ10610. Plasmids were introduced into the *ΔwhiB::apr* mutant SV7 by conjugation and their ability to restore sporulation was assessed both on solid and in liquid MYM medium. The *3xFLAG-[Gly_4_Ser]_3_-whiB* variant (C25S, C48S, C51S and C57S) plasmid pIJ10610 was also introduced into the *ΔwhiA ΔwhiB::apr* double mutant SV51. Additionally, a plasmid (pIJ10611), based on pIJ10750 was constructed to encode 3xFLAG-[Gly_4_Ser]_3_-WhiA driven from its own promoter (using the primer pair whiAcomp_82MCS_F/R and pIJ10601 as a template) and variant WhiB (C25S, C48S, C51S and C57S) driven by its own promoter (using the primer pair whiBcomp_82MCS_F/R and pIJ10608 as a template). This was then introduced into the *ΔwhiA ΔwhiB::apr* double mutant SV51. The SV7/pIJ10610 and SV51/pIJ10611 strains were subsequently used in ChIP-seq experiments.

**Western Blotting.** Samples of frozen mycelium, originating from 5 ml liquid MYM cultures, were resuspended in 0.4 ml ice-cold sonication buffer [20 mM Tris pH 8.0, 5 mM EDTA, 1 x EDTA-free protease inhibitors (Roche)] and sonicated (5x 15 sec on/15 sec off) at 4.5 micron amplitude. Lysates were then centrifuged at 16,000 xg for 15 min at 4˚C to remove cell debris. Total protein concentration was determined using the Bradford assay (Biorad). Equal amounts of total protein from each sample were loaded on a 12.5 % polyacrylamide SDS-PAGE gel. After electrophoresis, transfer was carried out to a Hybond-C Extra nylon membrane (Amerhsam Pharmacia Biotech) using the Invitrogen XCell II Blot system. For detection of 3xFLAG-[Gly_4_Ser]_3_-WhiA and 3xFLAG-[Gly_4_Ser]_3_-WhiB, anti-FLAG antibody (Sigma F4725) was used. In all cases the primary antibody was diluted in a ratio of 1:2500. For detection of WhiA, anti-WhiA polyclonal antibody was used at 1:2500. Proteins were visualised via an anti–rabbit IgG alkaline phosphatase secondary antibody (sigma A8025), diluted 1:5000 and detected directly on the membrane using the SigmaFast system (Sigma) that uses BCIP/NBT (5-Bromo-4-chloro-3-indolyl phosphate/Nitro blue tetrazolium) as a substrate.

**Scanning electron microscopy.** Colonies were mounted on the surface of an aluminum stub with optimal cutting temperature compound (Agar Scientific Ltd, Essex, UK), plunged into liquid nitrogen slush at approximately -210°C to cryopreserve the material, and transferred to the cryostage of an Alto 2500 cryotransfer system (Gatan, Oxford, England) attached to a Zeiss Supra 55 VP field emission gun scanning electron microscope (Carl Zeiss Ltd, Germany). The surface frost was sublimated at -95°C for 3 min before the sample was sputter coated with platinum for 2 min at 10 mA at below -110°C. Finally, the sample was moved onto the cryostage in the main chamber of the microscope, held at approximately -130°C, and viewed at 1.2 to 5.0 kV.

**Transmission electron microscopy.** Colonies were fixed in 2.5% (vol/vol) glutaraldehyde in 0.05 M sodium cacodylate, stained with osmic acid, and embedded in LR White resin according to the manufacturer’s instructions (The London Resin Co.). Sections were cut on a UC6 ultramicrotome (Leica Microsystems (UK) Ltd, Milton Keynes) and the grids were imaged using either a Jeol 1200EX or FEI Tecnai 20 TEM (FEI, The Netherlands), using an AMT digital camera (Deben, Bury ST Edmunds) to produce digital TIF files.

**Fluorescence microscopy.** Coverslips were placed gently on the surface of *S. venezuelae* colonies grown on solid MYM medium. Alternatively, coverslips was embedded at an angle in the agar and the strain inoculated along the inside edge of the coverslips prior to incubation. After incubation for 2-4 days, coverslips were removed, allowed to dry, soaked in 100% methanol for 1 minute and rinsed in water. 25 µg/ml Propidium Iodide and 50 µg/ml Wheat Germ Agglutinin (WGA) Alexa Fluor® 488, prepared in water was mixed and added to the surface of the coverslip (or the growth line) before incubation for 30 minutes in the dark. Next, the coverslips were rinsed in water, dried and placed onto the surface of a glass microscope slide with 10 µl of 20 % glycerol. Cells were then imaged using a Cairns CCD microscope at x100 magnification. Digital images were assembled using Image J and ADOBE photoshop software.

**REFERENCES**

1. **Gust B, Challis GL, Fowler K, Kieser T, Chater KF**. 2003. PCR-targeted *Streptomyces* gene replacement identifies a protein domain needed for biosynthesis of the sesquiterpene soil odor geosmin. Proc. Natl. Acad. Sci. U. S. A. **100:**1541–1546.
2. **Gust B, Chandra G, Jakimowicz D, Yuqing T, Bruton C, Chater KF**. 2004. Lambda red-mediated genetic manipulation of antibiotic-producing *Streptomyces*. Adv. Appl. Microbiol. **54:**107–128.
3. **Gregory MA, Till R, Smith MCM**. 2003. Integration site for *Streptomyces* phage ΦBT1 and development of site-specific integrating vectors. J. Bacteriol. **185:**5320–5323.
4. [**Fernández-Martínez LT**](http://www.ncbi.nlm.nih.gov/pubmed/?term=Fern%C3%A1ndez-Mart%C3%ADnez%20LT%5BAuthor%5D&cauthor=true&cauthor_uid=25403842)**,** [**Bibb MJ**](http://www.ncbi.nlm.nih.gov/pubmed/?term=Bibb%20MJ%5BAuthor%5D&cauthor=true&cauthor_uid=25403842). 2014 Use of the meganuclease I-SceI of *Saccharomyces cerevisiae* to select for gene deletions in actinomycetes. [Sci Rep.](http://www.ncbi.nlm.nih.gov/pubmed/?term=iscei+bibb) **4:**7100.
5. **Muth, G, Nubbaumer, B, Wohlleben, W, Puhler, A**. 1989. A vector system with temperature-sensitive replication for gene disruption and mutational cloning in streptomycetes. Mol. Gen. Genet. **219:**341–348.
6. [**Bush MJ**](http://www.ncbi.nlm.nih.gov/pubmed/?term=Bush%20MJ%5BAuthor%5D&cauthor=true&cauthor_uid=24065632)**,** [**Bibb MJ**](http://www.ncbi.nlm.nih.gov/pubmed/?term=Bibb%20MJ%5BAuthor%5D&cauthor=true&cauthor_uid=24065632)**,** [**Chandra G**](http://www.ncbi.nlm.nih.gov/pubmed/?term=Chandra%20G%5BAuthor%5D&cauthor=true&cauthor_uid=24065632)**,** [**Findlay KC**](http://www.ncbi.nlm.nih.gov/pubmed/?term=Findlay%20KC%5BAuthor%5D&cauthor=true&cauthor_uid=24065632)**,** [**Buttner MJ**](http://www.ncbi.nlm.nih.gov/pubmed/?term=Buttner%20MJ%5BAuthor%5D&cauthor=true&cauthor_uid=24065632). 2013. Genes required for aerial growth, cell division, and chromosome segregation are targets of WhiA before sporulation in *Streptomyces venezuelae*. [MBio.](http://www.ncbi.nlm.nih.gov/pubmed/?term=bush+whia) **4:**e00684–13.
7. **Stuttard C**. 1979. [Transduction of auxotrophic markers in a chloramphenicol-producing strain of *Streptomyces*.](http://www.ncbi.nlm.nih.gov/pubmed/438778) J. Gen. Microbiol. **110:**479–482.
